# Leaf Colour as a Signal of Chemical Defence to Insect Herbivores in Wild Cabbage (*Brassica oleracea*)

**DOI:** 10.1371/journal.pone.0136884

**Published:** 2015-09-09

**Authors:** Jonathan P. Green, Rosie Foster, Lucas Wilkins, Daniel Osorio, Susan E. Hartley

**Affiliations:** 1 Department of Biology, University of York, Heslington, York, United Kingdom; 2 School of Life Sciences, University of Sussex, Brighton, United Kingdom; Swedish University of Agricultural Sciences, SWEDEN

## Abstract

Leaf colour has been proposed to signal levels of host defence to insect herbivores, but we lack data on herbivory, leaf colour and levels of defence for wild host populations necessary to test this hypothesis. Such a test requires measurements of leaf spectra as they would be sensed by herbivore visual systems, as well as simultaneous measurements of chemical defences and herbivore responses to leaf colour in natural host-herbivore populations. In a large-scale field survey of wild cabbage (*Brassica oleracea*) populations, we show that variation in leaf colour and brightness, measured according to herbivore spectral sensitivities, predicts both levels of chemical defences (glucosinolates) and abundance of specialist lepidopteran (*Pieris rapae*) and hemipteran (*Brevicoryne brassicae*) herbivores. In subsequent experiments, *P*. *rapae* larvae achieved faster growth and greater pupal mass when feeding on plants with bluer leaves, which contained lower levels of aliphatic glucosinolates. Glucosinolate-mediated effects on larval performance may thus contribute to the association between *P*. *rapae* herbivory and leaf colour observed in the field. However, preference tests found no evidence that adult butterflies selected host plants based on leaf coloration. In the field, *B*. *brassicae* abundance varied with leaf brightness but greenhouse experiments were unable to identify any effects of brightness on aphid preference or performance. Our findings suggest that although leaf colour reflects both levels of host defences and herbivore abundance in the field, the ability of herbivores to respond to colour signals may be limited, even in species where performance is correlated with leaf colour.

## Introduction

Interactions between plants and their specialist herbivores are frequently the result of close co-evolutionary relationships [1]. Herbivores exert strong selection on their plant hosts leading to the evolution of a range of defences against herbivory [2]. Plant chemical defences can in turn have profound consequences for the herbivores that ingest them, and have selected for strategies by herbivores to minimize exposure to such toxins (e.g. [3, 4]). One strategy is for herbivores to avoid well-defended plants, but this depends on their ability to assess defence levels prior to attack or oviposition. It has long been recognized that plant volatiles convey information about host phenotype, including defence levels, and that olfactory cues are important for herbivores during host selection [5]. There has been less work on the importance of visual stimuli in host selection [6], but leaf coloration has attracted increasing interest following Hamilton and Brown’s [7] proposal that the autumn colours of trees have evolved to communicate host defence levels to aphids. Under this scenario, correlations between defence levels and leaf colour allow herbivores to use colour as a cue to defence [8]. Where herbivore responses to colour cues generate sufficient fitness benefits, plants may be selected to invest in leaf colour as a signal of their defensive commitment to herbivores [7]. More recently, this idea has been broadened to consider the role of leaf colour as a cue or signal of other host traits besides defence that are relevant to searching herbivores, for example nitrogen levels [1, 9, 10].

To date, research into the role of leaf colour in communicating defence levels to herbivores has focused largely on deciduous trees and aphids [1], following the observation by Hamilton and Brown [7] that interspecific variation in red autumn leaf coloration in trees was related to diversity of aphid herbivores. More recent studies focusing on intraspecific variation in leaf coloration in wild tree species have also reported correlations between leaf colour and aphid herbivory [9, 11–13]. However, progress in testing the hypothesis that leaf colour provides information about host defences to herbivores has been limited by the absence of data on the relationship between the colour and levels of chemical defence of leaves of plants in natural populations, together with information on how such defences impact herbivore abundance, preference and performance. Moreover, a fundamental requirement when determining the functional significance of leaf colour variation to herbivores is that such variation be measured according to the visual systems of the herbivores themselves [14], but to date most studies have sought to categorize such variation according to human rather than herbivore vision [7, 11–13], but see [9, 15]. The differences between the eyes of humans and insects mean that human colour vision does not provide an accurate account of the variation in leaf colour for aphids and other herbivorous insects [14].

This study examines the hypothesis that leaf colour provides information about host defences to herbivores by exploring the relationship between leaf colour, defence and herbivory in wild cabbage, *Brassica oleracea* L. (Brassicaceae). Interactions between wild cabbage and its specialist herbivores are an important model for studying the evolution and dynamics of plant-herbivore interactions (e.g. [16–21]). In *B*. *oleracea* and other Brassicaceae species, glucosinolates (hereafter GS) are a class of secondary metabolite that serve a crucial role in defence against insect herbivores, with numerous studies reporting negative impacts of both constitutive and induced GS on herbivore growth and survival (reviewed in [22]). Heritable variation within and between UK *B*. *oleracea* populations in GS expression has been linked to variation in herbivory pressure, suggesting that GS are under selection by herbivores [16, 19, 20] (but see [23]). Volatile cues associated with GS and their breakdown products are known to provide information about host defence to herbivores (e.g. [24]). In addition, wild *B*. *oleracea* also display striking variation in leaf coloration. To human observers, leaf hue falls either in a range from green to bluish green, or in a range from red to purplish-red [25]. Such variation may also be important in communicating host defence levels to herbivores during host searching. Support for the idea that colour accurately reflects defence levels comes from evidence for a link in other brassicas between the expression of leaf colour and GS [26], based on the presence of shared biochemical pathways for pigments and defences [27].

Here, we test whether *B*. *oleracea* leaf coloration predicts GS levels and herbivory by two specialist herbivores, the small white butterfly (*Pieris rapae*) and the cabbage aphid (*Brevicoryne brassicae*). Previous studies have provided evidence that high GS levels negatively affect the performance of both *P*. *rapae* [28–30] and *B*. *brassicae* [31, 32]. This is despite the fact that both species specialise on brassica so possess specific mechanisms to combat the toxic effects of GS: *P*. *rapae* is able to redirect GS hydrolysis away from the production of toxic isothiocyanates [3], while *B*. *brassicae* is able to sequester host GS [4]. These insects also use the presence of GS to identify brassica hosts on which to oviposit and feed [22, 33–35]. Though the presence of GS provides a useful marker for discriminating host from non-host species, the reduction in performance of both species associated with high GS levels suggests that to maximize offspring fitness *P*. *rapae* and *B*. *brassicae* could benefit from avoiding hosts with high GS levels. Indeed, there is evidence that herbivory by both species varies with GS content among wild host populations [19, 20].

For many insects, including aphids, colour preferences are thought to be based on an opponency mechanism that receives a positive input from the medium-wavelength sensitive (green) receptor and a negative input from the short-wavelength sensitive (blue) receptor [14, 36]. Pierid butterflies are relatively unusual among insects, however, in having long-wavelength sensitive (red) receptors, and *P*. *rapae* has two such receptors, termed ‘pale red’ and ‘deep red’, with peak sensitivities at 620 nm and 640nm respectively [37, 38]. Kelber [36, 39] proposed that comparison of red and green receptor inputs may allow butterflies to achieve greater discrimination among ‘green’ leaves than would be possible using green and blue receptors alone, and *P*. *rapae*’s two red receptors might permit even finer discrimination. In addition to chromatic cues provided by colour-opponency mechanisms, herbivores could potentially use achromatic (or brightness) cues to search for hosts, perhaps with green receptor output alone providing an achromatic channel for discriminating among leaves of differing brightness [40]. Aphids are thought to use brightness cues in host choice, though their importance relative to chromatic cues has been debated [14]. However, despite extensive work on the colour cues used by both butterflies and aphids in laboratory assays of host choice (e.g. [14, 36] and references therein), there has been little examination of their use within wild host populations (but see [9]).

The aim of this study is to determine whether variation in leaf coloration in a wild plant species signals defence levels to specialist herbivores. Combining detailed analysis of defence chemistry with measurement of leaf colour from the herbivores’ perspective, we first test whether variation in leaf colour among wild *B*. *oleracea* predicts levels of GS, a necessary precondition for the use of colour by herbivores to assess host defence levels. We then test for associations between leaf colour and abundance by *P*. *rapae* and *B*. *brassicae*. For *P*. *rapae* we relate herbivore presence to chromatic cues based on the relative excitation of the green receptor versus blue and red receptors. For *B*. *brassicae*, we relate herbivore presence to the relative excitation of the green and blue receptor; in addition, we explore responses to leaf brightness, based on excitation of the green receptor. In addition to defence levels, leaf colour may also provide information on other traits relevant to herbivores [1]. We therefore also determine correlations between herbivore abundance, leaf colour and traits including nitrogen levels, presence of flowers, and plant size and density.

While field surveys can provide information on associations between colour, chemistry and herbivory, they do not control for confounding effects of olfactory cues on host choice or for changes in plant GS levels in response to induction by herbivores [22]. Moreover, field data do not reveal the causal mechanisms underpinning associations between leaf colour, defence and herbivory. Thus, to examine correlations between leaf coloration and herbivory by *P*. *rapae* recorded in the field, and in particular to separate the effects of leaf colour from underlying chemistry on host selection and their subsequent growth and survival, we tested preference and performance under controlled greenhouse conditions. For *B*. *brassicae*, equivalent experiments were carried out to explore aphid preference and performance in relation to leaf brightness, which correlated most strongly with aphid abundance in the field.

## Materials and Methods

### Field Methods

#### Field surveys

Twelve spatially distinct populations of *B*. *oleracea* distributed over a range extending 500km along the south coast of England, UK were surveyed, three in each of four counties: Cornwall (50°10N, 5°42W; 50°12N, 5°42W; 50°10N, 5°41W), Devon (50°50N, 3°51W; 50°46N, 3°49W; 50°34N, 3°56W), Dorset (50°64N, 1°92W; 50°59N, 2°03W; 50°60N, 2°13W) and Kent (51°28N, 1°46E; 51°28N, 1°56E; 51°26N, 1°55E). These populations are located on maritime chalk cliffs and vary in size from several hundred to tens of thousands of plants. The *B*. *oleracea* populations surveyed in Dorset, Devon and Cornwall have been the focus of previous studies examining co-variation in GS levels and insect herbivory (e.g. [16, 19, 20, 23]). Permissions were obtained from the private land owners of all the field sites accessed in Cornwall, Dorset and Devon. In addition, permits were obtained for access to the study sites in Kent, which were on National Trust land. None of the field sampling at any of the sites involved endangered or protected species.

Each population was surveyed twice in 2010, once in early summer (20^th^ June -8^th^ July) and again in late summer (30^th^ August- 17^th^ September). During the first survey, 20–50 plants of a variety of ages were randomly sampled along a transect in each population (n = 548 plants in total). To permit identification of individual plants, each plant was photographed and labelled and a GPS reading (Garmin, Hampshire, UK) was taken. In the second survey, 304 plants identified in the first survey were re-located. During both surveys, each plant was searched thoroughly for insect herbivores and leaf samples were taken from each plant for analysis of leaf colour (see below). Lepidopteran larvae that were too small to be identified were reared to permit identification of later instars. *B*. *brassicae* counts were taken from the first survey, as aphids were absent at the time of the second survey, while *P*. *rapae* counts were taken from the second survey, as *P*. *rapae* was largely absent at the time of the first survey. *P*. *rapae* was found in all four counties, while *B*. *brassicae* was absent from populations in Kent. Analyses of *B*. *brassicae* herbivory thus used data from Cornwall, Devon and Dorset only. During the first survey, a random subset of plants were sampled from each population (4–15 plants, mean ± SD = 10.75 ± 3.5) for analysis of glucosinolate and C:N content (see below). Basal stem diameter, presence of flower heads and population density (measured as the number of other *B*. *oleracea* plants in the surrounding 2m^2^) were also recorded for each plant.

#### Leaf colour measurements

Colour measurements were taken from the third leaf from the top of each plant. Leaves were removed using scissors at the end of the day to control for variation in colour associated with changes in leaf water content throughout the day and stored in a white paper bag until colour readings were taken later in the evening. Reflectance spectra (300–700 nm) of leaves were recorded using an Ocean Optics USB2000 spectrometer. A small disc (diameter 0.5cm) was cut out from each leaf with a metal cutter, then transferred to the spectrometer using forceps. Samples were illuminated with a xenon light (Ocean Optics UV xenon lamp) and barium sulphate was used as a white standard. Colour measurements taken from multiple leaves (two new leaves, the focal leaf (the 3^rd^ leaf from the top), two mature leaves and two old leaves) in a subset of 18 plants confirmed that spectral data from the focal leaf were not significantly different from the mean values for all leaves. Hence, coloration of the focal leaf was representative of that of the plant as a whole.

**Fig 1:**
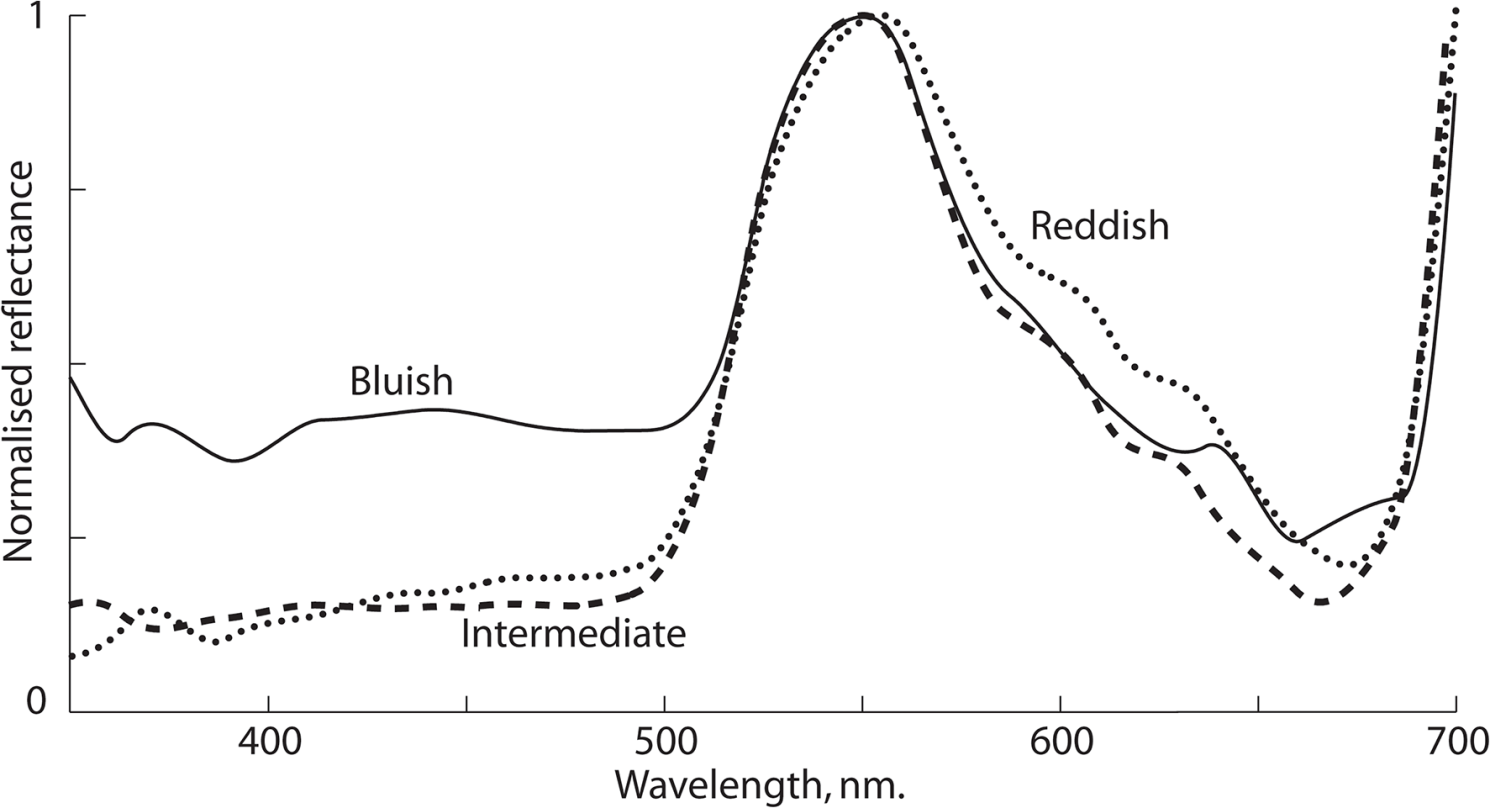
Reflectance spectra of three cabbage leaves of contrasting colour, normalised to the chlorophyll peak close to 550nm. *Solid line*: a bluish leaf with relatively high reflectance in the range 400-500nm, perhaps due to waxy epidermis; *Dashed line*: a leaf of intermediate coloration dominated by chlorophyll and probably appearing green to the human eye; *Dotted line*: a reddish leaf with relatively high reflectance above 550nm due the presence of long wavelength reflecting pigments. Spectra were recorded as described in the text, and then smoothed by fitting with a piecewise cubic function with 30 equal sections spanning the interval 300nm– 800nm was fitted to the spectra by minimising the total square deviation.

Leaf colour was modelled according to the spectral sensitivities of *P*. *rapae* and *B*. *brassicae* photoreceptors. For *P*. *rapae*, parameters used to model photoreceptor sensitivity curves were obtained from eye physiology data presented in [38]. Following evidence from other studies of butterfly colour vision [36, 39], we assume that colour discrimination is based on pairwise comparisons of green vs. red and green vs. blue receptor inputs. It is possible that additional spectral opponency mechanisms [41] could improve the animals’ colour vision, so this is a conservative prediction of performance. Leaf colour was analysed in terms of the ratios of (a) the blue to the green receptor quantum catch (henceforth, ‘B:G’); (b) the pale red to the green receptor quantum catch (‘PR:G ‘) and (c) the deep red to the green receptor quantum catch (‘DR:G’). To human observers, leaves with high B:G values will generally appear to have a bluish hue, while those with high R:G values will have a reddish hue. For *B*. *brassicae*, photoreceptor sensitivity curves were calculated based on peak receptor sensitivities at 325nm, 460nm and 530nm recorded for the aphid *Myzus persicae* [42], which are similar to that of *B*. *brassicae* (T. F. Döring, personal communication; see also [43]). Leaf spectra (see Fig 1 for typical examples) were analysed in terms of (a) the B to G quantum catch ratio as for *P*. *rapae* and (b) the green receptor quantum catch, an achromatic signal corresponding to the brightness of a leaf. Colour readings from leaf samples in the first survey were used in analyses of *B*. *brassicae* herbivory, while colour readings from the second summer survey were used in analyses of *P*. *rapae* herbivory. Values of B:G calculated from the *P*. *rapae* and *B*. *brassicae* models were extremely strongly correlated (r = 0.997, *P* < 0.0001).

#### Glucosinolate analysis

GS content was determined for 129 plants across the 12 sites. Discs (1cm in diameter) were punched from three randomly-chosen leaves on each plant. Leaf discs were pooled for each plant and placed in 5ml glass vials containing 1ml acetonitrile (HPLC-grade), which were then stored immediately on dry ice in a cryocontainer. In the lab, samples were stored at -80°C. GS were converted to desulphoglucosinolates on a Sephadex column and were identified using a combination of high-performance liquid chromatography (HPLC) and mass spectrometry following published methods [44]. In brief, freeze-dried plant material was ground to a coarse powder and 0.5mg placed in 2ml Eppendorf vials. GS were extracted with boiling 70% methanol and desulphatased using sulphatase solution (prepared from Sigma-Aldrich H-1 aryl sulphatase of *Helix pomatia*) on a DEAE Sephadex A-25 column (Fisher Scientific Ltd., Loughborough, UK). Desulphoglucosinolates were separated on a reverse-phase C18 Phenomenex column on HPLC with an acetonitrile water gradient. The desulphoglucosinolates were detected at 229nm using a UV detector. GS were identified using sinigrin hydrate (Sigma-Aldrich Company Ltd., Dorset, UK) as an external standard, which was desulphatased as above. GS identity was confirmed using mass spectrometry with ultra-high pressure liquid-chromatography (UPLC). Molecule identity from molecular mass was confirmed with the likely elemental composition function in the MassLynx software v. 4.1 (Waters Ltd., Hertfordshire, UK). To ensure retention times were consistent, we compared four replicate samples from a subset of plants. As with HPLC, sinigrin was used as an external standard. Five aliphatic GS (glucoiberin, gluconapin, glucoraphanin, progoitrin, sinigrin) and three indole GS (glucobrassicin, neoglucobrassicin and 4-methoxyglucobrassicin) were identified.

#### C:N analysis

Leaf samples from 179 plants across the 12 sites were dried to a constant weight for three days in a drying oven at 60°C. Whole leaf samples were then ground to a powder by pulverising at 50 oscillations for 30 seconds. Approximately 1.5mg of homogenised powder was weighed for C:N analysis. C:N content was determined by flash combustion and chromatographic separation, calibrated against a standard compound (C_26_H_26_N_2_O_2_S) using an elemental combustion system (Costech Instruments, Milan, Italy) [45].

### Herbivore preference and performance experiments

#### Plants

Three hundred wild *B*. *oleracea* plants were grown in 6-inch, 1.8L pots in John Innes potting compost no. 2 (John Innes Manufacturing Association, Berkshire, UK). Pots were spaced in a greenhouse at 15°C on a 16:8 light: dark regime. Plants were grown from a mixture of seeds collected from the three Dorset populations. Plants were watered as required and Hoagland solution [46] was applied weekly for the first month as a nutrient source. All plants were checked weekly for herbivores, which were removed. Leaf colour was measured for each plant three months after germination and modelled according to herbivore spectral sensitivities, as above.

#### *P*. *rapae* preference and performance

Subjects were offspring of 10 wild-caught individuals from sites in East Sussex. Following capture, adults were placed in a Perspex cage (50 x 60 x 70cm) for one week with a *B*. *oleracea* plant for oviposition. *Buddleia* flower heads provided a nectar source for the adults. Eggs were maintained in the same cage at 18°C and on a 16:8 light: dark regime. Larvae were reared on cut organic savoy cabbage (*B*. *oleracea var*. *sabauda*) purchased from a vegetable market. Emerging adults were fed *ad libitum* on 50% sugar solution. Adults had no contact with leaves of any kind, as previous host experience is known to influence the choice of oviposition sites [47]. Adults were allowed to fly freely in a greenhouse (380 x 310 x 240cm) to permit normal foraging and courtship behaviour.

**Fig 2:**
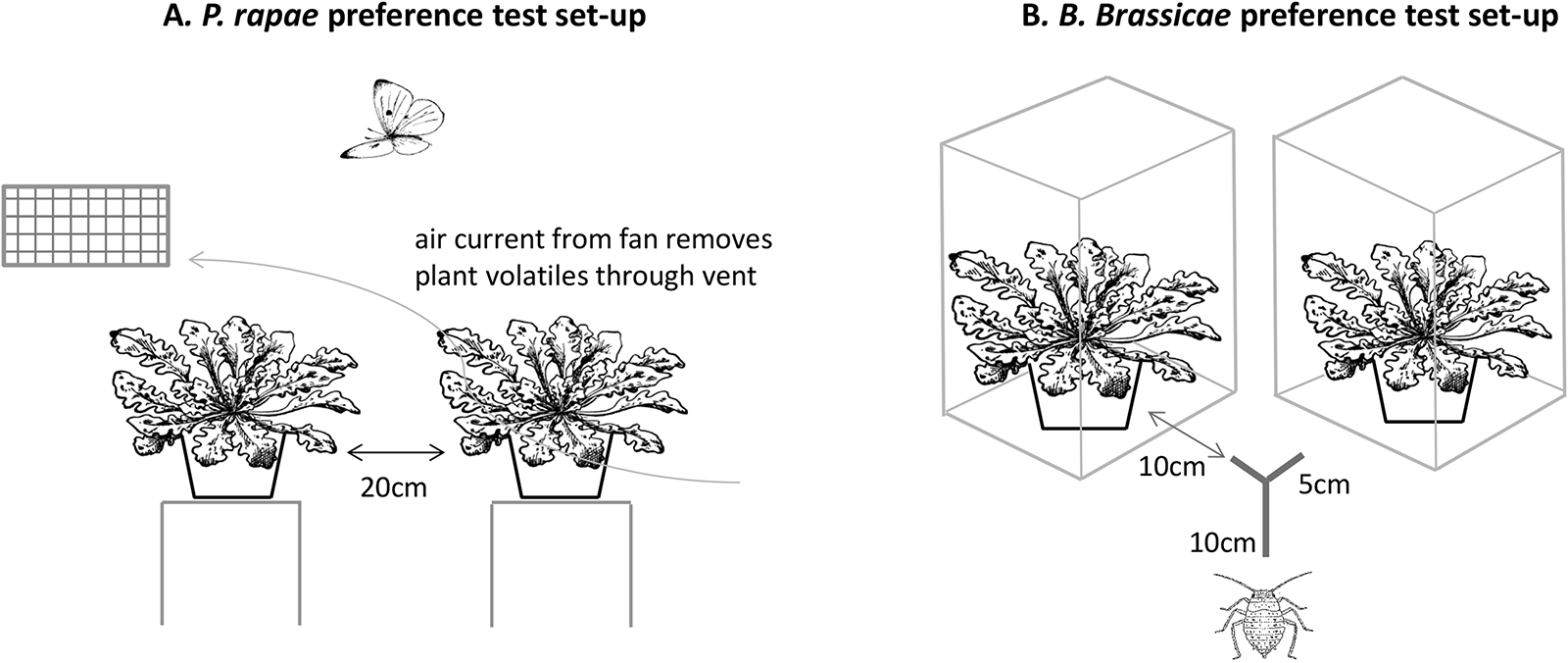
Experimental set-up of the herbivore preference experiments. (A) *P*. *rapae* responses to leaf colour were determined by recording landing responses of free-flying butterflies within a glasshouse. *B*. *oleracea* plants paired according to leaf colour were placed 20cm apart and on raised platforms 60cm from the ground. To remove plant volatiles, a fan blew air over the plants and out through a vent at the back of the glasshouse. (B) *B*. *brassicae* responses to leaf colour were determined by recording the choice made by aphids walking on a Y-stick. *B*. *oleracea* plants paired according to leaf colour were placed inside transparent plastic boxes against a plain white wall. Cling film was placed over the open side of the box facing the Y-stick to prevent aphids from detecting plant volatiles. At the start of each trial, an aphid was placed at the end of the 10-cm long arm of the Y-stick and allowed to walk towards the plants. Aphids were considered to have made a choice when they moved 1cm down one of the short 5cm arms.

In preference experiments, butterflies were given the choice of two plants differing in either the blueness (B:G ratio) or redness (DR:G ratio) of their leaves. From the 300 plants grown, the 30 plants with the highest B:G were paired with the 30 plants with the lowest B:G. Similarly, the 30 plants with the highest DR:G were paired with the 30 plants with the lowest DR:G. Sixteen plants were used in both the B:G and DR:G groups. To maintain consistent differences in ratio values between pairs of plants from the high and low groups, plants in each group were ranked according to their ratio values and the plants were then paired by rank (e.g. Rank 1 in high B:G group paired with Rank 1 in low B:G group etc.). Due to the need to pair plants of specific colour ranks, it was not possible to match plants precisely according to size, either in this experiment or in the aphid preference experiment described below. Tests were performed inside the greenhouse containing free-flying butterflies to ensure that host choice behaviour was as natural as possible. No artificial illumination was used. Pairs of plants were placed on raised platforms 20cm apart and 60cm from the ground (Fig 2A). Air was blown over the plants and outside the greenhouse through a vent using a fan to minimize detection of plant olfactory cues. The position of plants from the high and low groups in the greenhouse (left or right) was randomized across trials. At the start of each trial, both plants were presented simultaneously. A choice was recorded when a butterfly landed on one of the plants. Butterfly choices were recorded based on their decision to land rather than the decision to oviposit, as this is likely to be reinforced by contact with the leaf surface and the use of additional mechanical and/or olfactory cues. To prevent re-sampling of the same individuals, butterflies were captured after landing on the plants and placed in a cage. Individual choices were thus independent and the result of innate preferences, rather than preferences learnt during repeated exposure to hosts. Following a choice by one butterfly, the plant pair was removed and a new pair placed in position. A choice was made between all 30 B:G plant pairs and between all 30 DR:G plant pairs.

Following preference trials, larval performance was assessed on each plant in the B:G and DR:G groups. Two second-instar larvae were placed on each plant where they were contained within a cover of insulating garden fleece. Larvae were weighed at 9, 15, 20 and 24 days of age to record their growth. The area of leaf consumed for each plant was also estimated by eye. Plants were inspected daily for the presence of other herbivores, which were removed. Any larvae found to have died between 9 and 24 days were immediately replaced to ensure that levels of herbivore damage were equivalent across plants, thus minimizing variation in levels of induced defences resulting from differences in numbers of larvae between plants. Replacement larvae were maintained on organic savoy cabbage before use. Because replacement larvae were added to the plant on different dates to the original larvae, these individuals were not included in the growth rate calculations. Plants were checked daily for pupae, which were weighed and placed in individual pots. Emerging adults were sexed by examining wing spots. Relative growth rates for all larvae surviving to pupation were calculated as mg/mg/day [48]. The proportion of leaves remaining on each plant at the end of the trials ranged from 30–95%, indicating that larvae were not food-restricted during trials.

#### *B*. *brassicae* preference and performance

Aphid colonies were founded by individuals collected from non-experimental plants at the University of Sussex. Colonies were housed in ventilated Perspex boxes (60 x 50 x 70cm) at 20°C and on a 16:8 light regime. Colonies were maintained on Chinese cabbage (*B*. *rapa*) and subjects were naive to wild *B*. *oleracea*.

In preference experiments, adult apterates were given the choice of two plants differing in the brightness (G receptor quantum catch) of their leaves. From the 300 plants grown, the 30 plants with the brightest leaves were paired with the 30 plants with the dullest leaves following the procedure described above. Aphid preferences were tested in a choice test, comprising a 15cm Y-shaped cardboard stick, with each 5cm-long arm extending towards one of a pair of test plants positioned 10cm from the end of each arm at a 90° angle (Fig 1B). Plants were housed in transparent plastic boxes (56cm x 40cm x 30cm) with a cling film screen placed over the open face of the box facing the Y-stick, which permitted visual contact but prevented aphids from detecting plant volatiles. The boxes containing plants were placed in front of a white wall. The position of plants from the bright and dull groups (left or right) was randomized across trials. At the start of each trial, aphids were placed on the end of the Y-stick. Aphids were considered to have made a choice when they walked 1cm onto the right or left arm within 5 minutes of being placed on the end of the Y-stick. A new Y-stick was used in each trial to prevent individuals using cues left by other aphids when making a choice. The set-up was illuminated from above by a 20W Arcadia compact bird lamp (Arcadia Plc, Surrey, UK), designed to replicate the spectrum of sunlight between 300-700nm. Each aphid and plant pair was tested only once. Aphids used in the trials were starved for two hours prior to testing to increase motivation to find a host. A preliminary test of aphid responses to the choice of a plant vs. no plant confirmed that aphids in our set-up detected plants and moved towards them (binomial test, 23/32 aphids choosing the plant, P = 0.02).

Aphid performance was compared on 15 randomly-selected pairs of bright and dull plants used in the preference tests. Each plant was inoculated with 10 adult apterates, which were contained on plants within muslin bags. After five weeks, all aphids were removed, placed in alcohol and counted. Aphids were separated into alates and apterates. The number of dead aphids on each plant was also recorded.

### Statistical analysis

#### Field surveys

Statistical analyses were performed in R v.3.10.0 [49]. Correlations among colour measures and relationships between colour and plant size, density, presence of flowers and location were calculated using colour measurements collected from all 548 plants in the first survey. For correlations between colour and C:N, n = 179 plants and for correlations between colour and GS content, n = 129 plants. To control for multiple testing, we applied the Benjamini-Hochberg procedure [50] with the False Discovery Rate (FDR) set at 0.05. P values given in Tables 1 and 2 are unadjusted values.

**Table 1:** Correlations between chromatic and achromatic leaf cues and glucosinolate content for 129 *B*. *oleracea* plants sampled across all populations.

	B:G ratio	PR:G ratio	DR:G ratio	Brightness
**Total glucosinolates**	-0.06, 0.48	0.22, 0.01	**0.28, 0.002**	0.21, 0.02
**Total indoles**	-0.04, 0.68	0.20, 0.03	**0.26, 0.003**	0.21, 0.02
Glucobrassicin	-0.13, 0.15	0.23, 0.01	0.13, 0.14	-0.01, 0.95
Neoglucobrassicin	-0.02, 0.85	0.19, 0.03	**0.30, <0.001**	0.22, 0.01
4-Methoxyglucobrassicin	0.06, 0.48	0.01, 0.89	0.12, 0.18	0.05, 0.57
**Total aliphatics**	-0.07, 0.45	0.12, 0.18	0.03, 0.74	0.08, 0.38
Glucoiberin	-0.08, 0.37	0.16, 0.07	0.11, 0.23	0.15, 0.08
Gluconapin	0.07, 0.46	-0.17, 0.05	**-0.23, 0.009**	-0.10, 0.27
Glucoraphanin	-0.06, 0.51	0.18, 0.04	0.20, 0.02	0.16, 0.07
Progoitrin	0.07, 0.46	-0.07, 0.40	-0.01, 0.89	-0.06, 0.50
Sinigrin	-0.12, 0.17	0.13, 0.13	0.00, 1	0.17, 0.05

Values are Spearman’s rank correlation coefficients and associated *P*-values (ρ, *P*). Significant results following correction with the Benjamini-Hochberg procedure (FDR = 0.05) are in bold (*P* values shown are unadjusted).

**Table 2:** Relationship between chromatic and achromatic leaf cues and plant size, density, number of flowers and location.

Colour	Plant size	Density	Presence of flowers	C:N	County
**Brightness**	ρ = -0.08, *P* = 0.07	ρ = 0.05, *P* = 0.29	**F**_**1,546**_ **= 6.81, *P* = 0.009**	**ρ = 0.32, *P* < 0.0001**	**F**_**3,544**_ **= 12.24, *P* < 0.0001**
**B:G ratio**	ρ = -0.09, *P* = 0.03	ρ = 0.08, *P* = 0.10	**F**_**1,546**_ **= 17.61, *P* < 0.001**	**ρ = -0.29, *P* < 0.0001**	F_3,544_ = 1.16, *P* = 0.32
**PR:G ratio**	ρ = 0.07, *P* = 0.11	ρ = -0.10, *P* = 0.03	**F**_**1,546**_ **= 45.35, *P* < 0.0001**	**ρ = 0.37, *P* < 0.0001**	**F**_**3,544**_ **= 11.12, *P* < 0.0001**
**DR:G ratio**	ρ = -0.05, *P* = 0.27	ρ = -0.07, *P* = 0.16	**F**_**1,546**_ **= 16.23, *P* < 0.001**	ρ = -0.04, *P* = 0.60	**χ** ^**2**^_**3**_ **= 20.44, *P* = 0.0001**

Correlations between colour and plant size, density and C:N are Spearman’s rank correlations (ρ). Differences in colour between plants with and without flowers and between plants in different counties were analysed using ANOVA (F) and Kruskal-Wallis tests (*χ* ^*2*^). For correlations between colour and C:N, n = 179. For all other analyses, n = 548. Significant results following correction with the Benjamini-Hochberg procedure (FDR = 0.05) are in bold (*P* values shown are unadjusted).

To determine whether leaf colour was a significant predictor of herbivore presence, we analysed the effect of leaf colour on the presence or absence of *B*. *brassicae* (n = 398 plants) and *P*. *rapae* (n = 304 plants) in generalized linear mixed-effects models (GLMM) with binomial errors (glmer function in lme4 package [51]). Colour measures calculated for each herbivore were significantly correlated (see Results) and associated variance inflation factors (VIF) were all >10, indicating a high degree of multicollinearity [52], which prevented us from testing the effects of different colours in the same analysis. Instead, colour measures were fitted as single fixed effects in separate models. Spatial structuring was accounted for by fitting population as a random effect in each model, nested within county. Results for each colour measure are reported following removal of the fixed effect of colour from each model, with the change in deviance assessed using tabulated χ^2^ values. Because effects of colour measures on herbivore presence were tested in separate models, we used the Benjamini-Hochberg procedure to control the false discovery rate. In the complexity of the field, where other variables are not controlled for, it is reasonable to suppose that any effects of leaf colour on herbivore presence might be relatively small. Therefore, to increase our power to detect effects of individual colour measures on herbivore presence we selected a FDR of 0.1 when adjusting *P* values obtained from the GLMMs (see [53] for approaches to selecting FDR thresholds). *P* values given in the Results are unadjusted values.

In addition to testing for effects of leaf colour, we also tested for effects of GS and other plant phenotypic traits on herbivore presence, using a reduced data set consisting of plants for which measurements of leaf chemistry were available (n = 91 plants for *B*. *brassicae* and 79 for *P*. *rapae*). As above, we used GLMMs with binomial errors with spatial structuring accounted for by fitting population as a random effect, nested within county. For each herbivore species, we identified significant predictors of herbivore presence via backwards deletion of non-significant terms from a global model containing levels of the eight identified GS, together with plant size, density, C:N and the presence of flowers as fixed effect. First-order terms only were included in the global models, as preliminary data inspection indicated that two-way interactions were unlikely to be important in explaining herbivore presence and we had no a priori reason to suspect that such interactions might play an important ecological role in our system. Prior to running the analyses, we checked for multicollinearity among our predictors, which can produce misleading results when assessing significance of predictors via backwards deletion. VIF < 3 for all predictors contained with the global models, which we interpreted as implying a low degree of collinearity [52]. Backwards deletion of nonsignificant terms yielded a minimum adequate model, after which further removals led to significant (*P* < 0.05) increases in deviance, assessed using χ^2^ values. Significance levels are reported on the addition of non-significant terms and removal of significant terms from the minimum adequate model. Unless otherwise stated, only significant relationships between herbivory and levels of individual GS are reported. Model comparison using a forward selection procedure produced the same results for both herbivore species.

#### Herbivore preference and performance

Butterfly and aphid preferences were analysed with binomial tests (two-tailed). For analysis of *P*. *rapae* larval performance, mean values were calculated for pairs of larvae on the same plant. Larval performance between high vs. low B:G plants and high vs. low DR:G plants was compared using linear models (LM) with Normal errors. Colour ratio (high or low) was the predictor and time to pupation, pupal mass, leaf area eaten or relative growth rate as the response variable. Time to pupation and leaf area eaten were log-transformed to satisfy the normality assumption of the LM analysis. Aphid performance was analysed using LMs with the number of aphids (log-transformed) as the response variable and brightness (bright or dull) as the predictor. Proportions of total aphids that were alate, apterate or dead on bright and dull plants were analysed using generalized linear models (GLM) with quasibinomial errors to correct for overdispersion. For both LM and GLM analyses, significant (*P* < 0.05) terms were identified following removal of the predictor from each model, with the change in deviance assessed using tabulated F values.

## Results

### Colour, foliar chemistry and herbivory in the field

#### Correlations between coloration, foliar chemistry and other phenotypic traits

Among all plants surveyed, measures of leaf coloration based on *P*. *rapae* spectral sensitivities were significantly correlated. There was only a moderate positive correlation between PR:G and DR:G (Spearman’s ρ = 0.21, *P* < 0.001). B:G was strongly negatively correlated with PR:G (ρ = -0.81, *P* < 0.001) but positively correlated with DR:G (ρ = 0.34, *P* < 0.001). There was also a weak positive correlation between measures of brightness and B:G derived from the model of *B*. *brassicae* spectral sensitivities (ρ = 0.10, *P* = 0.02). Thus, plants that stimulated the *B*. *brassicae* green receptor (thus appearing brighter) also tended to stimulate the blue receptor more strongly relative to the green receptor.

Leaf coloration was significantly correlated with levels of several GS (Table 1). With the exception of gluconapin and progoitrin, DR:G and PR:G tended to be positively correlated with GS levels. Brightness had some tendency to be positively related to concentration of sinigrin and neoglucobrassicin, as well as with total concentration of indoles, but these correlations were not significant after applying the Benjamini-Hochberg correction. Leaf coloration was correlated with a number of other plant traits (Table 2). C:N was positively correlated with PR:G and brightness but negatively correlated with B:G. Flowering plants had higher DR:G and PR:G but lower B:G and brightness levels than plants without flowers. Finally, leaf R:G ratios were higher in more western counties, while leaf brightness was significantly greater in Dorset and Devon than in Cornwall or Kent.

#### *P*. *rapae* presence

**Fig 3:**
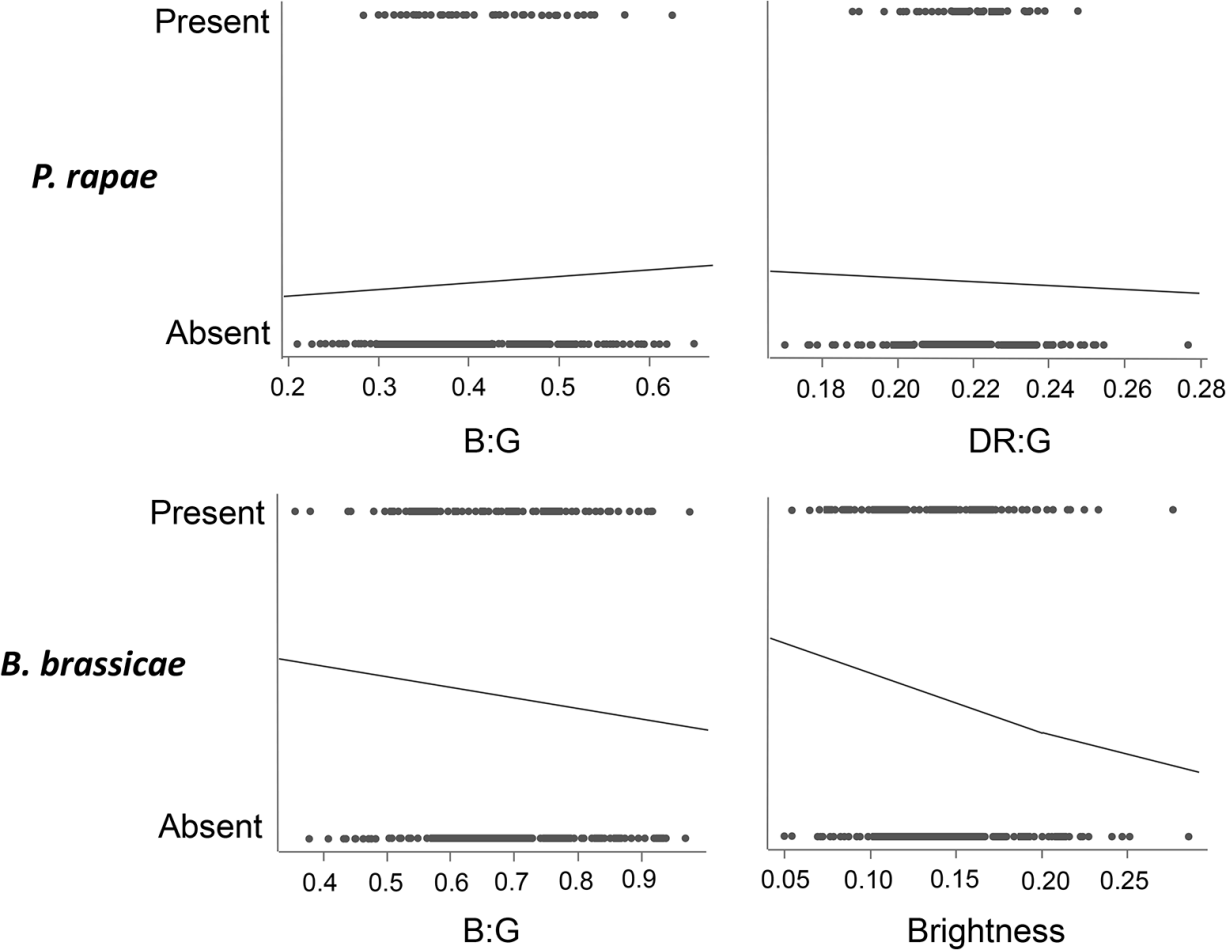
Relationship between *B*. *oleracea* leaf visual cues (B:G ratio, DR:G ratio and brightness) and the presence or absence of *P*. *rapae* and *B*. *brassicae*. Best fit lines were generated from GLMs with binomial errors with leaf colour as the predictor. *P*. *rapae* was more likely to be found on plants with higher B:G and lower DR:G, while *B*. *brassicae* was more likely to be found on plants with lower B:G and lower G (brightness).

*P*. *rapae* larvae were more likely to be present on plants with higher B:G (χ^2^_1_ = 4.11, *P* = 0.04; Fig 3) and lower DR:G (χ^2^_1_ = 4.33, *P* = 0.04; Fig 3). Larvae also tended to be more common among plants with lower PR:G, but this trend was not significant (χ^2^_1_ = 2.30, *P* = 0.08). Both B:G and DR:G remained significant predictors of *P*. *rapae* presence following correction using the Benjamini-Hochberg procedure. However, though B:G and DR:G were significant predictors of *P*. *rapae* presence, each explained only a very small proportion of the variance in *P*. *rapae* presence (marginal R^2^ values calculated according to [54] were < 5% in both cases), suggesting that any effect of colour on *P*. *rapae* abundance in the field is likely to be small.

*P*. *rapae* presence was not significantly predicted by levels of any GS, although there was a weak tendency for larvae to be more common on plants with lower neoglucobrassicin levels (χ^2^_1_ = 3.17, *P* = 0.08). Larvae also tended to be more common on plants with low C:N and on plants without flowers, though these trends were again nonsignificant (C:N: χ^2^_1_ = 3.69, *P* = 0.054; presence of flowers: χ^2^_1_ = 3.34, *P* = 0.07). Presence of *P*. *rapae* larvae was not related to plant size (χ^2^_1_ = 0.06, *P* = 0.80) or density (χ^2^_1_ = 0.31, *P* = 0.58).

#### *B*. *brassicae* presence

Aphids were more likely to be present on plants with lower B:G (χ^2^_1_ = 5.72, *P* = 0.02; Fig 3). Independently, aphids were also more likely to be present on duller plants (χ^2^_1_ = 12.41, *P* = 0.0004; Fig 3). Effects of both B:G and brightness remained significant following correction using the Benjamini-Hochberg procedure. However, though leaf colour was a significant predictor of aphid presence, the variance in aphid presence explained by B:G and brightness was again very small (marginal R^2^ < 5% in both cases). Thus, for *B*. *brassicae*, as for *P*. *rapae*, any effect of colour on abundance of aphids in the field is likely to be small.

In addition to effects of leaf colour, aphid presence was also associated with low levels of glucobrassicin (χ^2^_1_ = 6.38, *P* = 0.01), but did not vary significantly with any of the remaining GS. Aphids were also more common on plants with flowers (χ^2^_1_ = 6.96, *P* = 0.008), but their abundance did not vary with C:N (χ^2^_1_ = 2.22, *P* = 0.14) plant size (χ^2^_1_ = 1.50, *P* = 0.22) or density (χ^2^_1_ = 0.50, *P* = 0.48). Together, glucobrassicin levels and the presence of flowers explained 22% of the variance in aphid presence among the plants sampled for leaf chemistry (marginal R^2^ calculated for the combined fixed effects of glucobrassicin and presence of flowers according to [54]).

### Insect preference and performance

#### P. rapae

**Fig 4:**
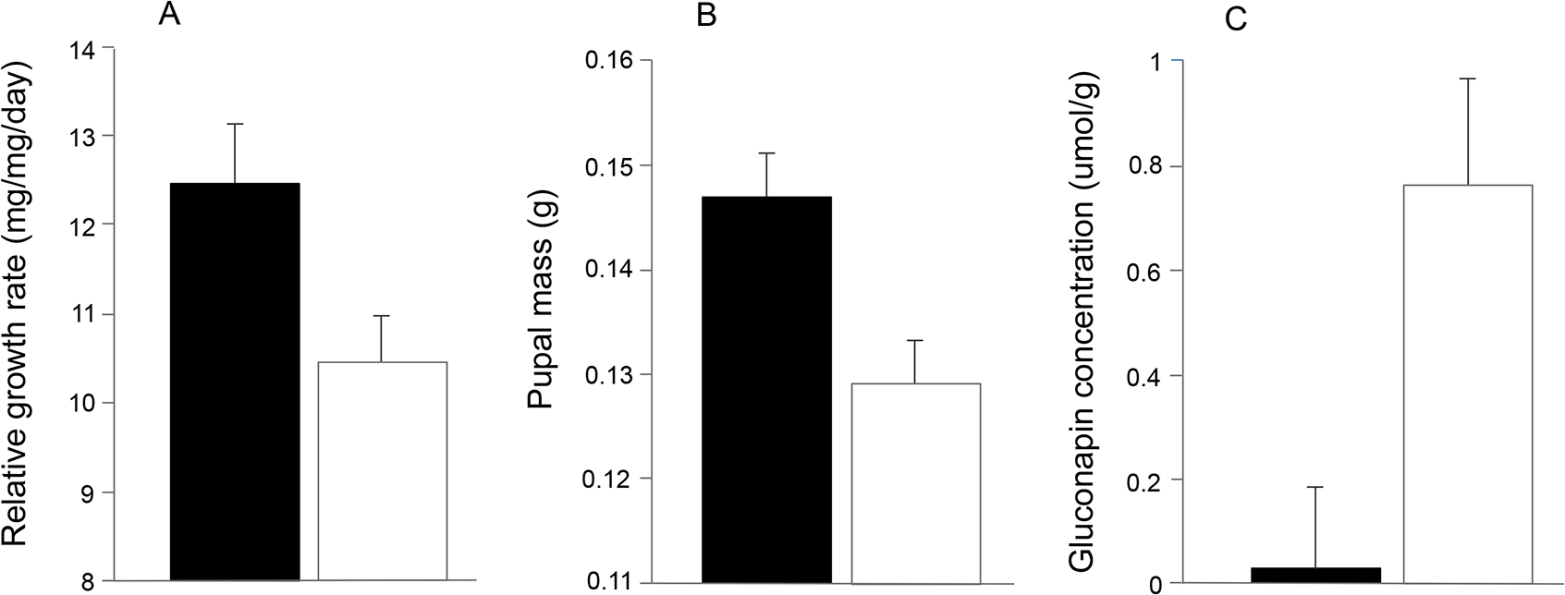
Differences in *P*. *rapae* performance and GS levels between high B:G (black bars) and low B:G (white bars) *B*. *oleracea* plants. A. Relative growth rates of larvae. B. Pupal mass (data show males and females). C. Concentration of the aliphatic GS gluconapin. Of the individual GS measured, only gluconapin levels were found to differ significantly between the bluest and least blue plants following Benjamini-Hochberg correction. Means ± SEM and results of statistical comparisons between high and B:G plants are presented in Table 2 (relative growth rate and pupal mass) and Table 3 (gluconapin concentration).

Butterflies showed no significant preference for either high vs. low DR:G plants (63% chose high DR:G, n = 30, *P* = 0.20) or high vs. low B:G plants (n = 30, 53% chose high B:G, *P* = 0.86). In performance trials, leaf consumption tended to be higher on low B:G and low DR:G plants, but these trends were not significant (Table 3). However, larval growth was found to vary significantly with plant colour. Growth rates were on average 20% higher and pupae 13% heavier on the high B:G vs. low B:G plants (growth rate: F_1,56_ = 5.48, *P* = 0.02; pupal mass: F_1,56_ = 10.10, *P* = 0.003; Fig 4A and 4B). The effect on pupal mass was more pronounced for females (17% increase, F_1,38_ = 10.62, *P* = 0.002) than for males (13.6% increase, F_1,29_ = 4.33, *P* = 0.05). By contrast, there was no difference in larval growth rates or pupal mass between high DR:G and low DR:G plants. Time to pupation did not differ between high vs. low B:G plants or between high vs. low DR:G plants (Table 3).

**Table 3:** Performance of *P*. *rapae* larvae on high vs. low B:G and DR:G plants.

	High B:G	Low B:G	F, *P*	High DR:G	Low DR:G	F, *P*
**Relative growth rate**	12.46 ± 0.69	10.42 ± 0.55	**F**_**1,56**_ **= 5.48, *P* = 0.02**	11.62 ± 0.54	11.25 ± 0.41	F_1,57_ = 0.33, *P* = 0.57
**Area of leaf eaten (cm** ^**2**^ **)**						
9 days	25.97 ± 7.11	29.19 ± 5.20	F_1,57_ = 0.22, *P* = 0.64	31.26 ± 5.64	35.71 ± 6.36	F_1,58_ = 0.67, *P* = 0.42
15 days	91.04 ± 15.42	123.3 ± 19.72	F_1,57_ = 1.27, *P* = 0.26	88.12 ± 13.77	95.09 ± 13.45	F_1,58_ = 1.05, *P* = 0.31
20 days	181.0 ± 25.09	240 ± 37.42	F_1,57_ = 0.96, *P* = 0.33	141.6 ± 18.38	171.8 ± 22.48	F_1,58_ = 1.75, *P* = 0.19
24 days	180.1 ± 23.95	253.4 ± 32.52	F_1,57_ = 3.24, *P* = 0.08	161.9 ± 19.04	197.7 ± 21.13	F_1,58_ = 2.44, *P* = 0.12
**Time to pupation (days)**	22.25 ± 0.75	23.25 ± 0.73	F_1,56_ = 0.96, *P* = 0.33	22.62 ± 0.76	21.93 ± 0.53	F_1,57_ = 0.43, *P* = 0.51
**Pupal mass (mg)**						
All pupae	146.9 ± 3.89	129.2 ± 4.00	**F**_**1,56**_ **= 10.10, *P* = 0.003**	140.9 ± 3.78	134.6 ± 3.36	F_1,57_ = 1.53, *P* = 0.22
Females	151.1 ± 4.80	129.2 ± 4.55	**F**_**1,38**_ **= 10.62, *P* = 0.002**	133.4 ± 6.30	133.6 ± 6.31	F_1,30_ = 0.00, *P* = 0.98
Males	142.3 ± 5.10	125.3 ± 6.52	**F**_**1,29**_ **= 4.33, *P* = 0.05**	141.2 ± 4.71	136.3 ± 4.04	F_1,35_ = 0.61, *P* = 0.44

Data are means ± SEM. F and *P*-values obtained from linear models (see main text). For analysis of leaf consumption, n = 29 high B:G, 30 low B:G, 30 high DR:G and 30 low DR:G plants. For analyses of growth rates, time to pupation and pupal mass, n = 28 high B:G, 30 low B:G, 30 high DR:G and 29 low DR:G plants (small differences in sample size between analyses are the result of several larvae failing to survive to pupation). Mean values were calculated for plants with >1 pupa. For analyses of female and male pupal masses, n = 40 females and 31 males for B:G plants and 32 females and 37 males for DR:G plants. Significant (*P* < 0.05) results are shown in bold.

Given the differences in larval performance between the high and low B:G plants, we analysed leaf samples collected before and after the performance experiment to determine whether differences in either constitutive or induced GS levels between the two plant groups could be responsible for the effects on larval growth and mass. Constitutive levels of total indoles did not differ between high and low B:G plants (Table 4). By contrast, the constitutive level of total aliphatics in low B:G plants was 171% higher than in high B:G plants, an effect driven by a large difference in the concentration of gluconapin and glucoraphanin (Fig 4C). All GS were induced above constitutive levels during the feeding trial (Wilcoxon matched-pairs test, aliphatics: W = 646, n = 37, *P* < 0.001; indoles: W = 697, *P* < 0.001), but there was no significant difference in levels of induced GS between high and low B:G plants following application of the Benjamini-Hochberg correction. There were no differences in any GS measures between high and low DR:G plants (Table 5). Induced levels of GS measured in the greenhouse plants were not significantly different from those recorded among plants in the field (Mann-Whitney test, W = 2370, n = 40 and 129, *P* = 0.40), suggesting that our experimental set-up was not a major driver of our findings.

**Table 4:** Constitutive and induced levels of glucosinolates (GS) in high vs. low B:G *B*. *oleracea* plants used in *P*. *rapae* performance tests.

	Constitutive levels		Induced levels		∆I-C
**Total glucosinolates**	1.50 ± 0.22 vs. 2.75 ± 0.36	**W = 231, *P* = 0.002**	7.44 ± 1.09 vs. 10.59 ± 4.25	W = 53, *P* = 0.85	W = 63, *P* = 0.35
**Total indoles**	0.87 ± 0.13 vs. 1.02 ± 0.19	W = 407, *P* = 0.68	4.96 ± 0.78 vs. 6.64 ± 3.97	W = 71, *P* = 0.12	W = 73, *P* = 0.09
Glucobrassicin	0.03 ± 0.00 vs. 0.04 ± 0.01	W = 342, *P* = 0.16	0.05 ± 0.01 vs. 0.05 ± 0.02	W = 53, *P* = 0.85	W = 68, *P* = 0.19
Neoglucobrassicin	0.12 ± 0.02 vs. 0.06 ± 0.02	W = 577, *P* = 0.03	1.57 ± 0.50 vs. 0.30 ± 0.11	W = 76, *P* = 0.05	W = 79, *P* = 0.03
4-Methoxyglucobrassicin	0.73 ± 0.12 vs. 0.92 ± 0.18	W = 375, *P* = 0.37	3.33 ± 0.80 vs. 6.27 ± 4.00	W = 55, *P* = 0.74	W = 61, *P* = 0.44
**Total aliphatics**	0.64 ± 0.19 vs. 1.74 ± 0.24	**W = 159, *P* < 0.001**	2.48 ± 0.98 vs. 3.95 ± 0.79	W = 26, *P* = 0.08	W = 40, *P* = 0.48
Glucoiberin	± 0.00 vs. 0.03 ± 0.02	W = 412, *P* = 0.70	0.07 ± 0.01 vs. 0.14 ± 0.06	W = 52, *P* = 0.91	W = 66, *P* = 0.24
Gluconapin	0.39 ± 0.16 vs. 1.14 ± 0.20	**W = 158, *P* < 0.001**	1.18 ± 0.75 vs. 2.28 ± 0.78	W = 30, *P* = 0.14	W = 42, *P* = 0.58
Glucoraphanin	0.01 ± 0.01 vs. 0.14 ± 0.04	W = 296, *P* = 0.02	0.05 ± 0.01 vs. 0.72 ± 0.40	W = 47, *P* = 0.85	W = 55, *P* = 0.74
Progoitrin	0.03 ± 0.01 vs. 0.14 ± 0.07	W = 397, *P* = 0.54	0.53 ± 0.17 vs. 0.37 ± 0.20	W = 71, *P* = 0.12	W = 67, *P* = 0.21
Sinigrin	0.20 ± 0.05 vs. 0.29 ± 0.06	W = 356, *P* = 0.23	0.64 ± 0.30 vs. 0.44 ± 0.12	W = 41, *P* = 0.53	W = 37, *P* = 0.34

For analysis of constitutive GS levels, n = 30 high and 29 low B:G plants. For analysis of induced GS levels, n = 10 high and 10 low B:G plants. ∆I-C = induced levels–constitutive levels within the same plant (n = 10 high and 10 low B:G plants). W = Mann-Whitney test. Significant results following correction with the Benjamini-Hochberg procedure (FDR = 0.05) are in bold (*P* values shown are unadjusted).

**Table 5:** Constitutive and induced levels of glucosinolates (GS) in high vs. low DR:G *B*. *oleracea* plants used in *P*. *rapae* performance tests.

	Constitutive levels		Induced levels		∆I-C
**Total glucosinolates**	1.91 ± 0.27 vs. 2.85 ± 0.33	W = 297, *P* = 0.04	9.17 ± 0.92 vs. 8.24 ± 1.10	W = 45, *P* = 0.42	W = 42, *P* = 0.61
**Total indoles**	0.88 ± 0.18 vs. 1.18 ± 0.19	W = 363, *P* = 0.28	4.11 ± 0.95 vs. 3.98 ± 0.92	W = 36, *P* = 1	W = 38, *P* = 0.89
Glucobrassicin	0.02 ± 0.00 vs. 0.05 ± 0.00	W = 266, *P* = 0.01	0.06 ± 0.02 vs. 0.06 ± 0.03	W = 34, *P* = 0.89	W = 40, *P* = 0.74
Neoglucobrassicin	0.24 ± 0.12 vs. 0.13 ± 0.05	W = 532, *P* = 0.14	1.69 ± 0.89 vs. 2.25 ± 1.02	W = 32, *P* = 0.74	W = 32, *P* = 0.74
4-Methoxyglucobrassicin	0.61 ± 0.12 vs. 0.99 ± 0.18	W = 353, *P* = 0.22	2.37 ± 0.67 vs. 1.66 ± 0.45	W = 41, *P* = 0.67	W = 44, *P* = 0.48
**Total aliphatics**	1.04 ± 0.02 vs. 1.68 ± 0.26	W = 310, *P* = 0.06	5.06 ± 1.05 vs. 4.26 ± 1.07	W = 43, *P* = 0.54	W = 44, *P* = 0.48
Glucoiberin	0.01 ± 0.00 vs. 0.01 ± 0.01	W = 454, *P* = 0.67	0.01 ± 0.01 vs. 0.02 ± 0.01	W = 31.5, *P* = 0.63	W = 24.5, *P* = 0.19
Gluconapin	0.71 ± 0.18 vs. 1.08 ± 0.21	W = 321, *P* = 0.09	3.52 ± 1.05 vs. 2.64 ± 0.85	W = 42, *P* = 0.61	W = 45, *P* = 0.42
Glucoraphanin	0.01 ± 0.01 vs. 0.09 ± 0.04	W = 394, *P* = 0.47	0.03 ± 0.01 vs. 0.03 ± 0.02	W = 41, *P* = 0.66	W = 47, *P* = 0.32
Progoitrin	0.07 ± 0.03 vs. 0.10 ± 0.06	W = 437, P = 0.99	0.77 ± 0.35 vs. 0.99 ± 0.55	W = 38.5, *P* = 0.85	W = 39, *P* = 0.81
Sinigrin	0.24 ± 0.05 vs. 0.39 ± 0.09	W = 399, P = 0.59	0.74 ± 0.20 vs. 0.58 ± 0.23	W = 45, *P* = 0.42	W = 49, *P* = 0.22

For analysis of constitutive GS levels, n = 30 high and 29 low DR:G plants. For analysis of induced GS levels, n = 9 high and 8 low DR:G plants. ∆I-C = induced levels–constitutive levels within the same plant (n = 10 high and 10 low DR:G plants). W = Mann-Whitney test. Significant results following correction with the Benjamini-Hochberg procedure (FDR = 0.05) are in bold (*P* values shown are unadjusted).

#### B. brassicae

Aphids showed no significant preference for either bright or dull plants (43% chose dull plants, n = 30, *P* = 0.58). There was no significant difference in the total number of aphids on bright and dull plants in the performance trials (1721 ± 387 vs. 1886 ± 388; F_1,28_ = 0.53, *P* = 0.47), or in the number of alate (90.5 ± 27.4 vs. 84.2 ± 15.9; F_1,28_ = 1.17, *P* = 0.29), apterate (1114 ± 263 vs. 1169 ± 241; F_1,28_ = 0.42, *P* = 0.52) and dead aphids (516 ± 124 vs. 633 ± 151; F_1,28_ = 0.69, *P* = 0.41). There was also no difference between bright and dull plants in the proportion of aphids that were alate (F_1,28_ = 0.47, *P* = 0.50) or died during the experiment (F_1,28_ = 0.93, *P* = 0.34).

## Discussion

Previous studies have shown that insect herbivores perceive naturally-occurring variation in autumn leaf colours [15] and that such variation predicts herbivory in the wild [9]. Our study, however, is the first to test the relationships between the variation in leaf colour in a wild plant species, analysed according to visual properties of the herbivores themselves, and both the presence and performance of key herbivore species, and the underlying defence chemistry of host plants. Three important findings derive from our field surveys. First, variation in leaf coloration among wild cabbages was associated with the presence of an hemipteran and a lepidopteran specialist herbivore (Fig 3). Second, variation in leaf coloration among cabbages predicted variation in levels of both aliphatic and indole GS. Third, levels of the indole glucobrassicin were negatively related to abundance of hemipteran herbivores. Furthermore, performance experiments under controlled conditions further established clear correlations between leaf coloration and larval growth and pupal mass in *P*. *rapae*, potentially mediated by the significant variation in concentrations of aliphatic GS in plants with different leaf coloration. It is significant that we were able to detect correlations between defence, coloration and herbivory in the field situation, despite factors such as spatial variation between plant populations and geographic regions, the range of herbivore species present at different densities, and the potential damage-induced increases in complex suites of GS over different time-scales [21, 22]. This suggests that these relationships may be sufficiently robust to serve as the basis for colour-mediated host use by herbivores in the wild.

### *P*. *rapae* herbivory in relation to plant coloration and defence

In the field, *P*. *rapae* larvae were more common on bluer plants (i.e. those with higher B:G values), though as noted above this effect was small. Results of experiments under controlled conditions found no evidence that adult butterflies preferred to oviposit on bluer plants, although larvae feeding on the bluest plants experienced faster growth and achieved a greater mass at pupation. Larval growth rate is known to determine vulnerability to parasitoids in *P*. *rapae*, with fast growth associated with reduced parasitism and increased survival [55]. Positive effects on larval growth may be a possible, though as yet untested, explanation for the increased presence of larvae on bluer plants in the field.

The increase in larval performance on bluer plants was matched by a significant reduction in constitutive levels of aliphatic GS compared with less blue plants. Previous studies have demonstrated that larval performance is impaired on hosts with high levels of aliphatic and indole GS [18, 29, 30]. GS-mediated effects on larval development thus offer a plausible explanation for the increased performance of larvae on bluer plants in our greenhouse experiments, although other, unmeasured differences between plants may also have impacted on larval performance. For example, in the field bluer plants were found to have lower C:N (Table 2), which has been shown to positively affect growth of *P*. *rapae* larvae [56]. The importance of N is also consistent with the negative trend between leaf C:N and larval abundance in our field study.

Whether GS-mediated performance effects explain the patterns of *P*. *rapae* herbivory recorded in the field is less clear. In line with the differences in GS levels between the blue plant groups in the greenhouse, bluer plants in the field tended to have lower levels of some GS; however, in the field the relationships between leaf blueness, GS levels and larval abundance tended to be nonsignificant. A number of other studies have also reported no correlation between *P*. *rapae* herbivory and GS levels within wild cabbage populations [23, 32]. In contrast, surveys by Newton et al. [19, 20] provide some evidence that *P*. *rapae* responds to variation in aliphatic GS, but the strength and direction of these responses were not consistent over years, potentially explaining why in a single field season we found no relationship between defence levels and herbivory. In addition, our ability to detect causal relationships between *P*. *rapae* herbivory and GS levels in the field was likely constrained to an extent by changes in GS profiles in response to a range of other factors, such as attack by other herbivore species and variation in the abiotic environment [17, 21].

The substantial increases in larval growth rate and pupal mass associated with feeding on bluer plants pose the question of why adults showed no preference for bluer plants. Though adult preferences often match larval performance [57], there are many exceptions [58,59]. One explanation could be that effects on growth and pupal mass associated with feeding on bluer plants may be too weak to generate selection on host searching behaviour. Although body size is considered to be a strong predictor of fecundity in insects [60], fecundity-mediated selection on body size may be weak if individuals seldom achieve their full reproductive potential [61]. Alternatively, negative effects associated with landing, and subsequently ovipositing, on bluer plants may counter selection for preferences for bluer hosts or else lead adults to adopt a risk-spreading strategy [62], searching out greener (lower B:G) hosts as well as their preferred bluer hosts. Such behaviour could potentially mask any innate preferences of adults for bluer hosts. If blueness is not used to identify suitable plants within host populations it may nevertheless enable butterflies to discriminate *B*. *oleracea*, which typically exhibit glaucous coloration, from non-host species exhibiting greener coloration, though this remains to be tested.

Primitively lepidopterans have UV, blue and green sensitive photoreceptors [63], but a number of lineages, including pierids, have evolved red receptors [38, 64, 65], potentially permitting greater discrimination between leaf greens than is possible using blue and green receptors alone [39]. Little is known, however, about the role of red receptors in host selection in the field, including the function of multiple red receptors in *P*. *rapae* [37]. Among wild cabbages, B:G, DR:G and PR:G were correlated but not perfectly so, implying that B:G, DR:G and PR:G could each provide independent information about host phenotype. In the field, cabbages with high DR:G experienced reduced herbivory by *P*. *rapae* (as for B:G, this effect was small, however) and there was a similar, though nonsignificant, relationship between herbivory and PR:G, consistent with the suggestion that excitation of the red receptor relative to the green receptor results in avoidance responses by butterflies [36, 39]. However, subsequent greenhouse experiments failed to identify a candidate mechanism linking herbivory and red leaf coloration, with no significant differences in landing preference or larval performance observed between the high and low red plant groups. Previous laboratory experiments have demonstrated avoidance of red coloration by *P*. *rapae* [47, 66], but these studies used artificial substrates that differed in their reflectance spectra to those of leaves and did not take butterfly spectral sensitivities into account. In the field, total GS levels and total indole levels were positively correlated with DR:G, and to a lesser extent with PR:G, which may account for the negative relationship between leaf redness and herbivory. However, we were unable to detect any such relationship among our greenhouse-grown plants. The mechanisms underpinning the association between *P*. *rapae* herbivory and leaf redness in the field are thus unclear and merit further investigation. In addition, there is little evidence from our field data for differing roles of DR and PR receptors, as relationships between both DR:G and PR:G and herbivory and GS levels were generally of a similar strength and direction. As with leaf blueness, it is possible that leaf redness plays a greater role in discrimination between host *B*. *oleracea* and non-host species, or among artificial cabbage varieties where differences in red coloration are greater (see e.g. [67, 68]).

### *B*. *brassicae* herbivory in relation to plant coloration and defence

*B*. *oleracea* leaf visual cues significantly predicted the presence of *B*. *brassicae* in the field, though in all cases effects of leaf colour were small. Aphid abundance varied most strongly with leaf brightness, with fewer aphids on plants with brighter leaves. In the field, brightness showed a positive correlation with total concentration of indole GS. Effects of indoles on phloem-feeding herbivores such as aphids are thought to be greater than those of aliphatics [22] and, in common with previous studies [31, 32], we found a negative relationship between aphid herbivory and levels of the indole glucobrassicin. High levels of indole GS thus provides a possible explanation for the negative correlation between leaf brightness and aphid herbivory. However, results of the greenhouse experiments provided no evidence that the greater numbers of aphids on dull plants in the field was due either to a preference for dull plants during initial host searching or to increased growth and survival of aphid colonies on dull plants.

In addition to leaf brightness, *B*. *brassicae* herbivory was also negatively associated with B:G. This latter finding is consistent with the idea that excitation of the blue receptor relative to the green receptor drives negative responses of aphids towards colour cues [15] and provides the first evidence that this opponency mechanism may function in host selection by *B*. *brassicae* under natural conditions. Both achromatic and chromatic cues have previously been considered to play a role in host choice by aphids, but their relative importance is still a matter of debate [14, 69]. In addition to visual cues, host selection by aphids is also known to be affected by a range of physical and chemical cues, including levels of host nutrients and defences, host water status, attractive or repellent host volatile emissions, epidermal or sieve element structure [69, 70]. Further investigation into how these factors correlate with leaf coloration in *B*. *oleracea* is needed to clarify the relative importance of chromatic and achromatic cues in determining patterns of herbivory by *B*. *brassicae* and to better understand the mechanisms underpinning association between aphid herbivory and leaf coloration in the field.

### Conclusions

Since Hamilton and Brown [7], the idea that leaf coloration may provide information about plant defence levels to herbivores has attracted considerable attention. Here, we have shown for the first time that defence levels in a wild plant species are correlated with chromatic and achromatic leaf cues that predict the abundance of specialist lepidopteran and hemipteran herbivores in the field. In the case of *P*. *rapae*, there is evidence that this association may be mediated by effects of leaf defensive chemicals on larval performance rather than by adult preferences for particular leaf colours. Our findings suggest that in this system, leaf colour is a reliable cue of host defence levels, but the ability of herbivores to respond to this cue may be constrained, despite herbivore performance parameters being correlated with leaf colour.
